# Musculoskeletal Corticosteroid Injection during COVID-19 Pandemic in Sabah: Is It Safe?

**DOI:** 10.1155/2021/8863210

**Published:** 2021-03-27

**Authors:** Mohamad Azwan Aziz, Redzal Abu Hanifah, Azmi Mohamed Mohd Nahar

**Affiliations:** ^1^Sports Medicine Department, University Malaya Medical Centre, Kuala Lumpur, Malaysia; ^2^Sports Medicine Unit, Queen Elizabeth Hospital, Kota Kinabalu, Sabah, Malaysia

## Abstract

Musculoskeletal corticosteroid injection is commonly used as an adjunct to help patients in pain management. In this current COVID-19 pandemic, many clinicians would differ from this treatment as steroid is considered an immunosuppressive drug and could risk the patient of developing severe adverse effects if contracting COVID-19. This is a retrospective study based in Sabah, Malaysia, examining the prevalence of COVID-19 infection following musculoskeletal corticosteroid injection from 1 December 2019 until 30 June 2020 in the sports medicine clinic and the orthopedic clinic. Patients who received musculoskeletal corticosteroid injection were called by telephone and asked about visits to the emergency department or government health clinic for influenza-like illness symptoms or severe acute respiratory infection that would require screening of COVID-19. Thirty-five patients who responded to the call were included, with mean ages of 47.9 years ± 15.1. 52% were male respondents, while 48% were female. 25% of them were diabetics, and 2.9% of them had a history of lymphoproliferative disorders. The mean pain score before injection was 6.74 ± 1.03 and after injection pain was 2.27 ± 1.63. In this study, there were 11.4% (*n* = 4) with minor complications of steroid injection, that is, skin discoloration. Nonetheless, there were no severe complications due to corticosteroids reported. There were no reported cases of COVID-19 among the respondents following corticosteroid injection. Musculoskeletal pain would affect a person's well-being and activities; thus, its management requires that careful consideration with risk-benefit analysis be made before administering musculoskeletal corticosteroid injection during COVID-19 pandemic.

## 1. Introduction

Musculoskeletal corticosteroid injection is a common procedure seen in a sports medicine clinic. The injection is used as an adjunct to treat various musculoskeletal diseases such as arthritic joint pain, bursitis, tendinopathy, and synovial inflammation. Robust studies have shown that corticosteroid injection helps patients control debilitating arthritic joint pain, improve the quality of daily living activities, improve range of motion, and an early return to work and, in certain patients, it helps delay or prevent surgical intervention [1]. Musculoskeletal corticosteroid injection is a safe procedure with a small risk of complications [1].

With the current public health emergency of COVID-19 outbreak, a careful decision of performing corticosteroid injection should be considered. Corticosteroid has an immunosuppressive effect. Numerous studies have reported that epidural steroid injection causes hypothalamic-pituitary axis (HPA) suppression from 4 to 12 weeks, and a single dose of intra-articular steroid injection could cause HPA suppression for up to 4 weeks [2–6]. Thus, in the early pandemic era, many professional national bodies have recommended against the usage of corticosteroid injection during this pandemic, as the fear of an asymptomatic patient infected with SARS-CoV-2 could put them at increased risk of an adverse outcome from the virus if they had received the injection [7]. The rising concern for use of injectable corticosteroids in musculoskeletal care comes from established guidelines from World Health Organization for systemic steroid use in patients under treatment for active COVID-19. According to the recommendation by the interim guidelines of the World Health Organization during early pandemic, use of systemic corticosteroids is not recommended for treating severe COVID-19 infection as no good evidence shows the benefits of steroid usage and there was persuasive evidence of adverse short- and long-term harm [8]. In a retrospective study by Sytsma et al., they have shown an increased risk of contracting influenza infection during the influenza season in patients given a corticosteroid injection [9]. However, this study was unable to demonstrate the timeline between time of receiving corticosteroid injection, time of influenza vaccine delivered, and time of contracting influenza infection. In early global COVID-19 pandemic, the British Society for Rheumatology and the British Society of Skeletal Radiologists published a recommendation against musculoskeletal steroid injection except for severe debilitating pain despite optimum conservative therapy [10, 11].

We are concerned with the clear wholesale withdrawal of musculoskeletal steroid injection as a beneficial treatment option for patients who have severe and painful musculoskeletal conditions during the COVID-19 pandemic. As patients suffer and surgical management options seem months away, surgical alternatives are unlikely to be available, and it is good practice to consider conservative management with injection before committing to surgery. The latest guidelines by the National Health Service (England) on 16 June 2020, were for musculoskeletal steroid injections to be considered for severe symptoms after failed physical rehabilitation and splinting [12]. Currently, the evidence of increased risk of systemic infection including COVID-19 following intra-articular and periarticular corticosteroid injection is limited. A recent study by Chang et al. reported no significant elevation in the risk of developing COVID-19 infection following steroid injection compared to the general population [13]. McKean et al. also demonstrate a low incidence of COVID-19 following corticosteroid injection with no major adverse outcomes [14]. We hypothesize that there will be a very low risk of developing COVID-19 infection following musculoskeletal steroid injection. The study aims to determine the prevalence of COVID-19 infection following musculoskeletal steroid injection during the pandemic in Sabah, Malaysia.

## 2. Methods and Materials

### 2.1. Study Design

This study was conducted retrospectively from 1 December 2019 until 30 June 2020 during the COVID-19 pandemic in the state of Sabah, Malaysia. The date was chosen with respect to the first reported case of COVID-19 by the World Health Organization on 31 December 2019. Numerous studies conclude that a single dose of intra-articular steroid injection can cause HPA suppression of up to 4 weeks [2–6]. Thus, this study begins at 1 December 2019. All medical case notes were retrieved. Patients included in the study were those that had sought treatment in the sports medicine clinic and the orthopedic clinic in Queen Elizabeth Hospital and Queen Elizabeth 2 Hospital, Sabah, with presentation of inflammatory arthritis, non-inflammatory arthritis, bursitis, tenosynovitis, epicondylitis, and carpal tunnel syndrome, and were treated with corticosteroid injection with further follow-up given for 2 weeks. Demographic data including name, identification number, age, ethnicity, medical comorbidity, and diagnosis for clinical visitation were included in this study. The date of injection was recorded. Patients were excluded from this study if they had intramuscular steroid injections due to nonmusculoskeletal issues, intra-articular or soft tissue injections without using steroids, or incomplete data in their case notes. For injection over the finger, a dose of 20 mg triamcinolone was used per body part, while, in other parts of the body, a dose of 40 mg triamcinolone was used per body part. Once the patients were identified and included in this study, the investigator would call the patients, and the purpose of the study was explained. When consent was given by the patients, they were asked about any visit to the emergency department, government clinic, or private hospital for COVID-19 screening with symptoms of Influenza-Like Illness (ILI) or Severe Acute Respiratory Infection (SARI).

This study was conducted in compliance with ethical principles outlined in the Declaration of Helsinki and Malaysian Good Clinical Practice Guideline with approval from the Medical Research Ethics Committee.

### 2.2. Statistical Analysis

Descriptive analysis was done for this research. The participant's baseline characteristics, types of musculoskeletal corticosteroid injection, complication, and improvement were recorded in this study.

## 3. Results

Out of 52 intra-articular/periarticular injection procedures, 11 procedures did not use corticosteroids as an agent and 6 patients had incomplete or missing data.  Table 1 shows the demographic characteristics of 35 patients enrolled in this study, with ages ranging from 27 to 85 years and a mean age of 47.9 ± 15.1 years. 52% were male and 48% were female. 25% are diagnosed with diabetes mellitus and 2.9% had a history of lymphoproliferative disorders. 17% of the patients were above 65 years of age.

All 35 patients required musculoskeletal corticosteroid injection as an adjunct for pain management. In Table 2, the most common sites of injections were knee (*n* = 15, 42.9%) and shoulder (*n* = 7, 20%). The most common indication for the injection was for knee osteoarthritis (Table 3). All patients received triamcinolone as steroid injection with 2% lignocaine as local anaesthetic. 34.3% of the patients had a corticosteroid injection with additional prolotherapy of Dextrose 50%. Three patients received 20 mg triamcinolone over fingers, 2 patients received 80 mg triamcinolone in bilateral knees in the same day, and the rest received 40 mg triamcinolone.

In this study, only 11.4% had minor complications of steroid injection as in Table 4. All were skin discoloration. Nonetheless, there were no serious or severe complications due to corticosteroids. There were no complications reported for local anaesthesia. The mean pain score before injection was 6.74 ± 1.03, and in the following injection the mean pain score was 2.27 ± 1.63 one week after the procedure (Table 5). None of the patients reported symptoms of ILI, SARI, or confirmed cases of COVID-19 following musculoskeletal corticosteroid injection.

## 4. Discussion

There were major concerns in administering musculoskeletal steroid injections during COVID-19 pandemic. Many clinicians believe that administering musculoskeletal corticosteroids risks the patient to adverse outcomes of COVID-19 [7]. The key finding in our study showed that all patients who had musculoskeletal corticosteroid injection do not have symptoms of Influenza-Like Illness or Severe Acute Respiratory Syndrome, which would mandate for both nasopharyngeal and oropharyngeal swabs real-time polymerase chain reaction (rt-PCR) for SARS-CoV-2 in government health facilities. This could be partly due to the prevalence of COVID-19 in Sabah being relatively low (9.7 per 100 000 population), with an efficient public health system in promoting good preventive methods for spreading COVID-19 such as wearing masks in public, avoiding crowded places, social distancing, and frequent hand-washing [15].

34.3% of patients in our study had at least one comorbidity and 83% were below 65 years of age. Systma et al. have examined the outcome of corticosteroid injection on risk of contracting influenza [9]. In Systma et al.'s study, a total of 15,068 joint injections have been analyzed [9]. There was an increased relative risk of 1.5 for vaccinated subjects developing influenza after an intra-articular corticosteroid using methylprednisolone (79%), betamethasone (11%), and triamcinolone (10%). The corticosteroid dose was 66 mg with a range of 40–120 mg [9]. In our study, there were zero rates of COVID-19 infection. Our study used triamcinolone (100%). According to Systma et al.'s study (2018), women below 65 years of age have a higher risk of contracting infection [9]. They examined the risk of contracting influenza infection after patients received influenza vaccine [9]. High influenza rate in the younger population (2.28% (*n* = 46 of 2015) versus 1.18% in elderly patient >65 years (*n* = 33 of 2789); *P* < 0.001) is possibly due to high dose vaccine received by the geriatric population [9]. This was the first study to identify the risk of contracting infection following corticosteroid injection during pandemic seasons. However, this study was unable to show the timeline between time of receiving the vaccine to the time that the patient received steroid injection, and the time when patients contracted the infection.

A recent study by Chang et al. also demonstrated no increased risk of COVID-19 infection following corticosteroid injection [13]. Out of 66 subjects who completed follow-up in 45 ± 22 (19–83) days after injection, 1 subject was positive for COVID-19 infection following injection. The rate of infection was 1.52% [13]. The subject had mild symptoms that did not require any hospitalization [13]. A larger study with 504 steroid injections had a 2.7% rate of infection, of which 1.1% were asymptomatic. There were no severe adverse outcomes to COVID-19 infection reported in the study [14]. Compared to our study, where we have zero infection rates, the rate of infection of COVID-19 is still low with no severe adverse outcomes following steroid injection.

A careful consideration with risk-benefit analysis should be applied before administering corticosteroid injection. In this study, there was improvement seen in pain score in all patients. The incidence of skin discoloration in this study was higher than that in Stephens et al.'s study (2001) of <1% [1]. This may be due to a small sample size in our study. There were 4 patients (11.4%) who had skin discoloration in this study, which resolved after a few months. Robust cases have reported incidence of hypopigmentation after triamcinolone injection, which is the type of steroid (100%) used in this study [16–18]. The mechanism of hypopigmentation is not well understood, but biopsy study showed that there is a decrease in melanocyte function rather than actual loss of melanocytes [16–18]. One of the possible reasons for high cases of hypopigmentation was the use of high dose of steroid (40 mg) in small soft tissue such as wrist and fingers. A careful explanation regarding the possible adverse effects of corticosteroid injection, including skin pigmentation, should be highlighted before the procedure.

Robust studies have shown the usage of corticosteroids in managements of COVID-19. A single-blind randomized controlled clinical trial on the usage of pulse methylprednisolone showed a significant survival improvement in severe COVID-19 infection [19]. RECOVERY trial also demonstrated a significant beneficial survival effect of systemic corticosteroids in severe and critically ill COVID-19 patients requiring respiratory support [20]. A prospective meta-analysis of seven randomized clinical trials by the WHO Rapid Evidence Appraisal for the COVID-19 Therapies Working Group also showed a mortality benefit when using corticosteroids compared to placebo [21]. This shows that corticosteroids are useful and have a significant benefit in management of COVID-19.

Although this is a retrospective descriptive study, it is important to conduct further research of the risk of contracting respiratory infection with corticosteroid injection during influenza outbreaks and the COVID-19 pandemic. A further risk analysis study is required to strengthen the knowledge for improvement in delivering pain management in patients who are in need. The major limitation of our study is that the true number of COVID-19 infections remains unknown in this patient cohort, as all subjects did not undergo a screening test during this period as there were no symptoms. There is high possibility of the presence of asymptomatic COVID-19 patients in this population study. This is because the Ministry of Health in Malaysia is focusing on a targeted screening approach of COVID-19 rather than mass testing. One of the other possible reasons why this study has no COVID-19 infection following corticosteroid injection is the small sample group and low prevalence of COVID-19 in Malaysia. The situation of COVID-19 in Malaysia as of 11 July 2020 was 8704 positive cases, 26.7 per 100 000 population [15]. In Sabah, there were 379 positive cases, 9.7 per 100 000 population [15]. Our aim in the future is to examine a larger population in Malaysia on the complex interaction between the host immune response, steroid injection, and risk of COVID-19 infection. Other limitations that we identify during this study are that, due to a small sample size, we were unable to risk stratifying patients who are at higher risk of contracting COVID-19 infection. Sytsma et al. identified that female patients below 65 years of age have a higher risk of contracting influenza infection [9]. Future direction of research should focus on bigger populations to examine the risk of contracting COVID-19 following musculoskeletal steroid injections. Artificial intelligence technology has created its way in the research field to examine big databases. For example, Zhang et al. discussed the use of artificial intelligence technology in improving the patient care with acute lung injury and acute respiratory distress syndrome [22]. Similar things could be applied during COVID-19 pandemic era to examine a bigger dataset.

## 5. Conclusion

In this study, there was no COVID-19 infection following intra-articular and soft tissue corticosteroid injection. There was no reported case of major complications following corticosteroid injection. 11.4% of patients reported skin discoloration at the site of injection. Thus, we suggest a careful consideration of risk-benefit analysis before administering musculoskeletal corticosteroids injection to patients who would gain the benefit of corticosteroid's effect on pain and inflammation. However, due to the small sample size, we could not conclude that musculoskeletal steroid injection brings no risk of contracting COVID-19 infection.

## Figures and Tables

**Table 1:** Demographic data of patients who received musculoskeletal steroid injections.

Characteristic	Range (years)	Mean (years)	SD
Age	27–85	47.9	15.1

Race		*n*	%
	Malay	5	14.3
	Chinese	14	40
	Bumiputera, Sabah	16	45.7

Comorbid			
	No comorbids	23	65.7
	Diabetes mellitus	9	25
	Hypertension	6	17
	Gout	1	2.9
	Lymphoproliferative disorder	1	2.9

*Note*. Age is described in mean ± SD. Other demographic data are described in numbers and percentage.

**Table 2:** Characteristics of musculoskeletal steroid injections.

Characteristics of steroid injection		*n*	%
Area of injection			
	Ankle	2	5.7
	Elbow	2	5.7
	Finger	3	8.6
	Knee	15	42.9
	Shoulder	7	20
	Spine	1	2.9
	Wrist	5	14.3

Number of joint injections per day per person			
	Single joint injection	33	94.3
	Multiple joint injections	2	5.7

Type of steroid			
	Triamcinolone	35	100

Type of anaesthesia			
	2% lignocaine	35	100

Additional prolotherapy			
	With prolotherapy	12	34.3
	Without prolotherapy	23	65.7

**Table 3:** Indications for musculoskeletal steroid injections.

Diagnosis	*n*	%
Acromioclavicular osteoarthritis	1	2.9
Carpal tunnel syndrome	1	2.9
De Quervain tenosynovitis	4	11.4
Frozen shoulder	2	5.7
Hallux valgus	1	2.9
Knee osteoarthritis	14	40
Pes anserinus	1	2.9
Ankle impingement syndrome	1	2.9
S1 radiculopathy	1	2.9
Subacromial impingement	4	11.4
Supraspinatus tendinitis	1	2.9
Tennis elbow	2	5.7
Trigger finger	2	5.7

**Table 4:** Complications following musculoskeletal injections.

Complications	*N*	%
Patients presenting to hospital, diagnosed as COVID-19 after injection	0	0
Skin discoloration	4	11.4
No reported complications	31	88.6

**Table 5:** Outcome of musculoskeletal steroid injections in terms of pain score before and after procedures.

Pain score	Mean	SD
Preinjection pain score	6.74	1.03
Postinjection pain score	2.27	1.63

## Data Availability

The data used to support the findings of this study are included within the supplementary information file without name and identification number information to protect patient's privacy based on Medical Research Ethics Committee.
