# Supplementary material for: Musculoskeletal Corticosteroid Injection during COVID-19 Pandemic in Sabah: Is It Safe?
Source: Adv Orthop. 2021 Mar 27;2021:8863210. doi: 10.1155/2021/8863210 (PMC8006753; doi:10.1155/2021/8863210)
Supplement: Supplementary Materials — Tables S1–S4. [file 8863210.f1.docx]

**Data Collection of Retrospective study of musculoskeletal corticosteroid injection during COVID-19 pandemic in Sabah**

**Supplementary table 1: Demographic Data**

| Number | Gender | Race | Age | Any Underlying Comorbid | Diagnosis |
| --- | --- | --- | --- | --- | --- |
| 1 | female | Chinese | 78 | T-Cell lymphoproliferative disease | trigger finger |
| 2 | female | Kadazan | 52 | nil | supraspinatus tendinitis |
| 3 | female | india | 49 | nil | knee osteoarthritis |
| 4 | male | Chinese | 35 | nil | knee osteoarthritis |
| 5 | male | Chinese | 64 | nil | trigger finger |
| 6 | male | Kadazan | 38 | nil | knee osteoarthritis |
| 7 | male | Brunei | 34 | nil | knee osteoarthritis |
| 8 | male | Melayu | 32 | nil | knee osteoarthritis |
| 9 | male | Chinese | 65 | dm | frozen shoulder |
| 10 | male | Kadazan | 42 | Diabetes/hypertension | knee osteoarthritis |
| 11 | female | bugis | 48 | hypertension | subacromial impingement |
| 12 | male | Bisaya | 33 | nil | knee osteoarthritis |
| 13 | male | Melayu | 35 | nil | right ankle impingement |
| 14 | male | Bajau | 56 | Gout | knee osteoarthritis |
| 15 | male | Chinese | 65 | Diabetes/hypertension | acromionclavicular osteoarthritis |
| 16 | male | Chinese | 46 | Diabetes | subacromial impingement |
| 17 | female | Bajau | 44 | Diabetes/hypertension | hallux valgus |
| 18 | female | Chinese | 63 | Diabetes | knee osteoarthritis |
| 19 | male | Sino Kdzn | 37 | Nil | knee osteoarthritis |
| 20 | female | Chinese | 50 | Nil | subacromial impingement |
| 21 | female | Chinese | 50 | Nil | tennis elbow |
| 22 | female | Melayu | 37 | Nil | de quirvain tenosynovitis |
| 23 | female | Melayu | 37 | Nil | carpla tunnel syndrome |
| 24 | female | Melayu | 77 | Diabetes/hypertension | subacromial impingement |
| 25 | male | Chinese | 52 | Nil | de quirvain tenosynovitis |
| 26 | female | Brunei | 27 | Nil | pes anserinus |
| 27 | male | Chinese | 30 | Nil | tennis elbow |
| 28 | male | Chinese | 60 | Nil | de quirvain tenosynovitis |
| 29 | male | Kadazan | 41 | Nil | knee osteoarthritis |
| 30 | female | Kadazan | 37 | Nil | de quirvain tenosynovitis |
| 31 | female | Murut | 29 | Nil | S1 radiculopathy |
| 32 | male | Kadazan | 39 | Nil | knee osteoarthritis |
| 33 | female | Chinese | 70 | Diabetes/hypertension | frozen shoulder |
| 34 | female | Bisaya | 42 | Diabetes | knee osteoarthritis |
| 35 | female | Chinese | 85 | Nil | knee osteoarthritis |

**Supplementary table 2: Injection steroid**

| Number | Type of injection | Location of injection | Medication | Dosage/joint | Number of injection in one day | Usage of analgesic/name | Usage of prolotherapy | Date of injection |
| --- | --- | --- | --- | --- | --- | --- | --- | --- |
| 1 | periarticular | finger | Triamcinolone | 20mg | single | 2% lignocaine | no | 01/06/2020 |
| 2 | intraarticular | shoulder | Triamcinolone | 40mg | single | 2% lignocaine | yes | 1/24/2020 |
| 3 | intraarticular | knee | Triamcinolone | 40mg | single | 2% lignocaine | no | 02/03/2020 |
| 4 | intraarticular | knee | Triamcinolone | 80mg | multiple | 2% lignocaine | yes | 2/17/2020 |
| 5 | periarticular | finger | Triamcinolone | 20mg | single | 2% lignocaine | yes | 2/17/2020 |
| 6 | intraarticular | knee | Triamcinolone | 40mg | single | 2% lignocaine | no | 2/17/2020 |
| 7 | intraarticular | knee | Triamcinolone | 40mg | single | 2% lignocaine | no | 2/17/2020 |
| 8 | intraarticular | knee | Triamcinolone | 40mg | single | 2% lignocaine | yes | 2/17/2020 |
| 9 | intraarticular | shoulder | Triamcinolone | 40mg | single | 2% lignocaine | no | 2/24/2020 |
| 10 | intraarticular | knee | Triamcinolone | 40mg | single | 2% lignocaine | no | 2/24/2020 |
| 11 | periarticular | shoulder | Triamcinolone | 40mg | single | 2% lignocaine | no | 2/27/2020 |
| 12 | intraarticular | knee | Triamcinolone | 80mg | multiple | 2% lignocaine | yes | 2/27/2020 |
| 13 | periarticular | ankle | Triamcinolone | 40mg | single | 2% lignocaine | no | 2/28/2020 |
| 14 | intraarticular | knee | Triamcinolone | 40mg | single | 2% lignocaine | yes | 03/02/2020 |
| 15 | periarticular | finger | Triamcinolone | 20mg | single | 2% lignocaine | no | 03/12/2020 |
| 16 | intraarticular | shoulder | Triamcinolone | 40mg | single | 2% lignocaine | yes | 6/15/2020 |
| 17 | periarticular | ankle | Triamcinolone | 40mg | single | 2% lignocaine | no | 6/25/2020 |
| 18 | intraarticular | knee | Triamcinolone | 40mg | single | 2% lignocaine | no | 6/29/2020 |
| 19 | intraarticular | knee | Triamcinolone | 40mg | single | 2% lignocaine | yes | 2/27/2020 |
| 20 | intraarticular | shoulder | Triamcinolone | 40mg | single | 2% lignocaine | no | 26.12.2019 |
| 21 | periarticular | elbow | Triamcinolone | 40mg | single | 2% lignocaine | no | 26.12.2019 |
| 22 | periarticular | wrist | Triamcinolone | 40mg | single | 2% lignocaine | no | 10.12.2019 |
| 23 | periarticular | wrist | Triamcinolone | 40mg | single | 2% lignocaine | no | 18.12.2020 |
| 24 | intraarticular | shoulder | Triamcinolone | 40mg | single | 2% lignocaine | no | 21.1.20 |
| 25 | periarticular | wrist | Triamcinolone | 40mg | single | 2% lignocaine | no | 19.2.2020 |
| 26 | periarticular | knee | Triamcinolone | 40mg | single | 2% lignocaine | no | 27.2.2020 |
| 27 | periarticular | elbow | Triamcinolone | 40mg | single | 2% lignocaine | no | 14.4.2020 |
| 28 | periarticular | wrist | Triamcinolone | 40mg | single | 2% lignocaine | no | 12/09/2019 |
| 29 | intraarticular | knee | Triamcinolone | 40mg | single | 2% lignocaine | yes | 12/09/2019 |
| 30 | periarticular | wrist | Triamcinolone | 40mg | single | 2% lignocaine | no | 12/16/2019 |
| 31 | intraarticular | spine | Triamcinolone | 40mg | single | 2% lignocaine | no | 16/12/2019 |
| 32 | intraarticular | knee | Triamcinolone | 40mg | single | 2% lignocaine | no | 12/26/2019 |
| 33 | intraarticular | shoulder | Triamcinolone | 40mg | single | 2% lignocaine | yes | 12/30/2019 |
| 34 | intraarticular | knee | Triamcinolone | 40mg | single | 2% lignocaine | yes | 12/30/2019 |
| 35 | intraarticular | knee | Triamcinolone | 40mg | single | 2% lignocaine | yes | 12/31/2019 |

**Supplementary table 3: outcome and complications**

| Number | Pain score prior to injection | Pain score on follow up 2 weeks post injection | complications | History of URTI | History of Covid-19 screening (any swab taken) | History of admission treated as influenza like illness or SARI | History of quarantine |
| --- | --- | --- | --- | --- | --- | --- | --- |
| 1 | 8 | 0 | Nil | Nil | Nil | Nil | Nil |
| 2 | 6 | 2 | Nil | Nil | Nil | Nil | Nil |
| 3 | 6 | 4 | Nil | Nil | Nil | Nil | Nil |
| 4 | 6 | 4 | Nil | Nil | Nil | Nil | Nil |
| 5 | 8 | 0 | skin discoloration | Nil | Nil | Nil | Nil |
| 6 | 8 | 4 | Nil | Nil | Nil | Nil | Nil |
| 7 | 7 | 5 | Nil | Nil | Nil | Nil | Nil |
| 8 | 7 | 3 | Nil | Nil | Nil | Nil | Nil |
| 9 | 8 | 3 | Nil | Nil | Nil | Nil | Nil |
| 10 | 5 | 5 | Nil | Nil | Nil | Nil | Nil |
| 11 | 8 | 2 | Nil | YES (April) | Nil | Nil | Nil |
| 12 | 6 | 4 | Nil | Nil | Nil | Nil | Nil |
| 13 | 7 | 4 | Nil | Nil | Nil | Nil | Nil |
| 14 | 6 | 3 | Nil | Nil | Nil | Nil | Nil |
| 15 | 7 | 3 | Nil | Nil | Nil | Nil | Nil |
| 16 | 6 | 1 | Nil | Nil | Nil | Nil | Nil |
| 17 | 6 | 1 | skin discoloration | Nil | Nil | Nil | Nil |
| 18 | 6 | 3 | Nil | Nil | Nil | Nil | Nil |
| 19 | 6 | 2 | Nil | Nil | Nil | Nil | Nil |
| 20 | 7 | 2 | Nil | YES (May) | Nil | Nil | Nil |
| 21 | 8 | 0 | Nil | Nil | Nil | Nil | Nil |
| 22 | 7 | 0 | Nil | Nil | Nil | Nil | Nil |
| 23 | 4 | 2 | skin discolouration | Nil | Nil | Nil | Nil |
| 24 | 7 | 3 | Nil | Nil | Nil | Nil | Nil |
| 25 | 8 | 0 | Nil | Nil | Nil | Nil | Nil |
| 26 | 9 | 0 | Nil | Nil | Nil | Nil | Nil |
| 27 | 7 | 0 | Nil | Nil | Nil | Nil | Nil |
| 28 | 7 | 0 | Nil | Nil | Nil | Nil | Nil |
| 29 | 6 | 3 | Nil | Nil | Nil | Nil | Nil |
| 30 | 8 | 0 | skin discoloration | Nil | Nil | Nil | Nil |
| 31 | 6 | 4 | Nil | Nil | Nil | Nil | Nil |
| 32 | 7 | 4 | Nil | Nil | Nil | Nil | Nil |
| 33 | 6 | 2 | Nil | Nil | Nil | Nil | Nil |
| 34 | 6 | 3 | Nil | Nil | Nil | Nil | Nil |
| 35 | 6 | 3 | nil | Nil | Nil | Nil | Nil |

**Supplementary table 4: Exclusion data**

- Missing data/incomplete data: 6 patients
- No corticosteroid used: 11 patients
